# A Subtle Interplay Between Three Pex11 Proteins Shapes *De Novo* Formation and Fission of Peroxisomes

**DOI:** 10.1111/j.1600-0854.2011.01290.x

**Published:** 2011-10-20

**Authors:** Anja Huber, Johannes Koch, Friedrich Kragler, Cécile Brocard, Andreas Hartig

**Affiliations:** 1 University of Vienna, Max F. Perutz Laboratories, Center for Molecular Biology, Department of Biochemistry and Cell BiologyDr. Bohr-Gasse 9, A-1030 Vienna, Austria; 2 Department II, Max Planck Institute of Molecular Plant PhysiologyGolm, Germany

**Keywords:** fatty acid consumption, inheritance assay, membrane elongation, membrane proteins, organelle biogenesis, peroxisomes, PEX11, PEX25, PEX27, proliferation

## Abstract

The organization of eukaryotic cells into membrane-bound compartments must be faithfully sustained for survival of the cell. A subtle equilibrium exists between the degradation and the proliferation of organelles. Commonly, proliferation is initiated by a membrane remodeling process. Here, we dissect the function of proteins driving organelle proliferation in the particular case of peroxisomes. These organelles are formed either through a growth and division process from existing peroxisomes or *de novo* from the endoplasmic reticulum (ER). Among the proteins involved in the biogenesis of peroxisomes, peroxins, members of the Pex11 protein family participate in peroxisomal membrane alterations. In the yeast *Saccharomyces cerevisiae*, the Pex11 family consists of three proteins, Pex11p, Pex25p and Pex27p. Here we demonstrate that yeast mutants lacking peroxisomes require the presence of Pex25p to regenerate this organelle *de novo*. We also provide evidence showing that Pex27p inhibits peroxisomal function and illustrate that Pex25p initiates elongation of the peroxisomal membrane. Our data establish that although structurally conserved each of the three Pex11 protein family members plays a distinct role. While *Sc*Pex11p promotes the proliferation of peroxisomes already present in the cell, *Sc*Pex25p initiates remodeling at the peroxisomal membrane and *Sc*Pex27p acts to counter this activity. In addition, we reveal that *Sc*Pex25p acts in concert with Pex3p in the initiation of *de novo* peroxisome biogenesis from the ER.

A consequence of the modular organization of the eukaryotic cytoplasm into membrane-bound organelles is an increase in the efficiency of metabolic processes. Such arrangement provides tailored microenvironments for chemical reactions in the cell. The modular organization is associated with a subtle equilibrium between proliferation and degradation of all subcellular compartments. For proliferation, organellar membranes are remodeled in a restricted area to accommodate altered protein and lipid composition, leading to polarization of the organelle. Polarizing events are usually initiated by the insertion of morphogenic proteins which alter the membrane curvature and sustain protrusion of this membrane (1,2). A consequence thereof is local membrane instability, which is ultimately resolved by fission. Accordingly at the onset of peroxisome proliferation, extensions form at the peroxisomal membrane (3,4). The number of peroxisomes per cell increases through growth and division of pre-existing peroxisomes (5) or, when required, peroxisome biogenesis is initiated *de novo* at the endoplasmic reticulum (ER) (6–11). These processes are controlled and executed by peroxins (PEX proteins) which act to maintain the peroxisomal compartment thereby sustaining cellular homeostasis.

Conceptually, peroxisome proliferation can be divided into five steps. Initially, proliferation needs to be spatiotemporally defined at the peroxisomal membrane (step 1), leading to polarized growth of the membrane, its protrusion (step 2) and elongation (step 3). Step 4 comprises the import of matrix proteins into the elongated area and recruitment of the fission machinery coinciding with constriction of the organellar membrane. Finally, scission and separation into individual peroxisomes (step 5) is carried out by fission factors shared with mitochondria (3,12,13).

Among the peroxins implicated in peroxisome proliferation, Pex11 proteins directly influence the elongation of the peroxisomal membrane (3,13–15). We explored the role of the Pex11 proteins employing a panoply of *Saccharomyces cerevisiae* mutants with peroxisome biogenesis defects. Previous work focusing on members of the Pex11 family in yeast, Pex11p, Pex25p and Pex27p suggested that each plays a different role in peroxisome function (16–19). However, comprehensive insight regarding their interplay and specific function in forming new peroxisomes is still missing. Here we present data demonstrating that Pex11p acts to maintain the peroxisomes in a metabolically active state and to proliferate already existing peroxisomes. Based on *in vivo* studies we established that Pex25p serves as an initiating factor in the process of membrane proliferation. In addition, we showed that after the complete loss of peroxisomes, Pex25p is the main factor of this family responsible for the regeneration of the organelle. Our data also support a model in which Pex27p competes with Pex25p and negatively affects peroxisomal function.

## Results

### Heterologous Pex11 proteins can substitute for PEX11 in *S. cerevisiae*

**Figure 1:**
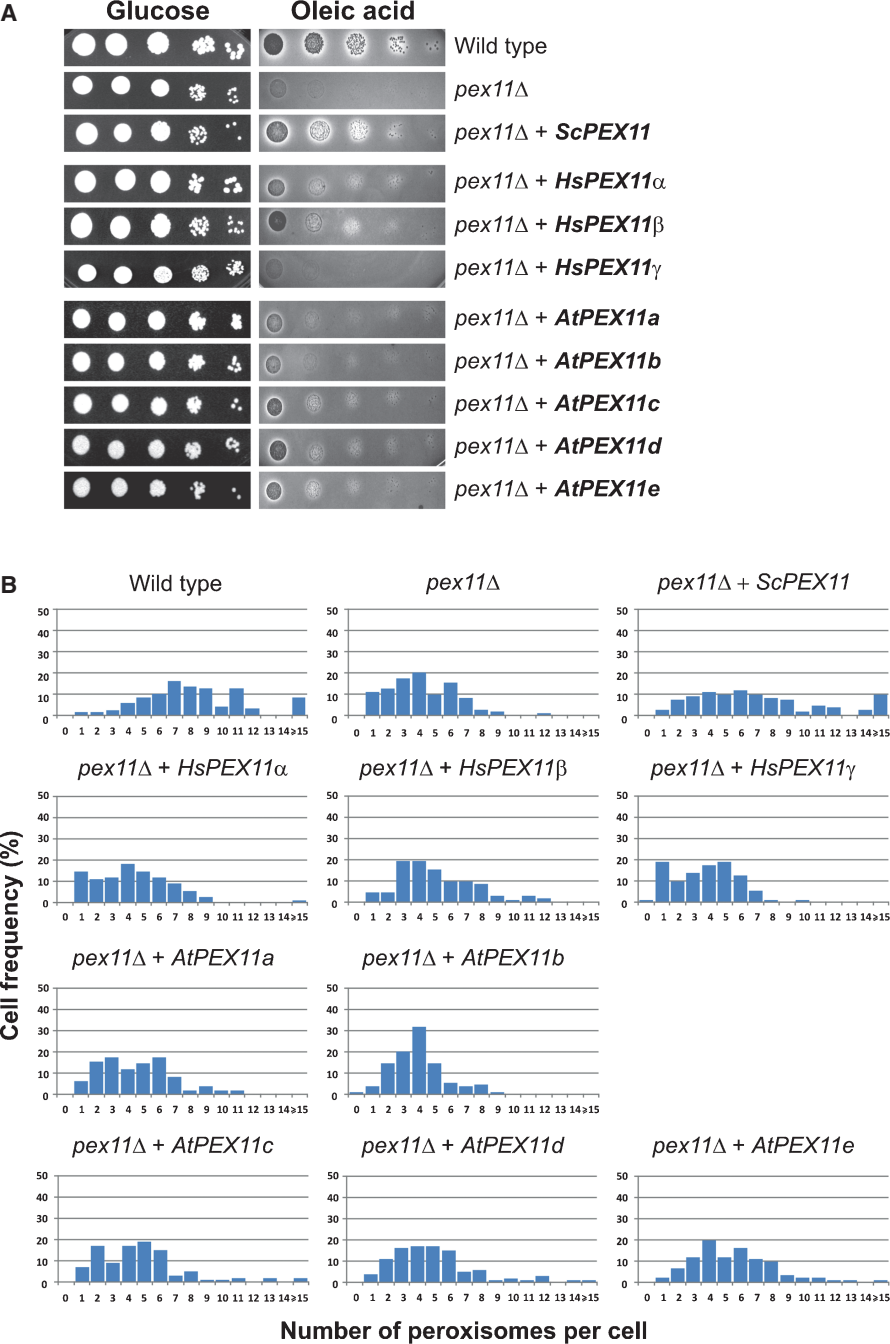
Heterologous Pex11 proteins can compensate for the peroxisomal defect of *pex11*Δ yeast cells A) Yeast cells lacking the *PEX11* gene were transformed with plasmids expressing Pex11 proteins from different organisms, grown to logarithmic phase in medium containing 0.3% glucose and 10-fold serial dilutions were spotted onto glucose or oleic acid-containing agar plates. Oleate utilization was monitored by the formation of a clear zone (halo assay). B) Peroxisomes were visualized through mCherry-px (red channel) in cells, described in (A), incubated for 16 h in oleic acid-containing medium. For each strain, the fluorescent dots were counted in 100 non-budding cells. The histograms illustrate the frequency of cells with a distinct number of peroxisomes.

Yeast cells lacking *PEX11* contain few enlarged peroxisomes and are unable to utilize fatty acids as a carbon source (16,17,20). Conversely, overexpression of *PEX11* leads to the occurrence of many small peroxisomes. Hence, there seems to be a correlation between the number, the size and the function of peroxisomes. Generally, cells lacking Pex11 proteins present reduced peroxisomal function (21–23). To explore the evolutionary conservation of Pex11 protein function, we examined the effect of expressing human (PEX11α, PEX11β, PEX11γ; 24–26) or plant (PEX11a to e; 22) Pex11-proteins in *pex11*Δ yeast cells. We assessed the ability of the cells to utilize oleic acid and determined the number of peroxisomes per cell (Figure 1). The human PEX11α and PEX11β and the plant PEX11c, PEX11d and PEX11e complemented the oleate utilization defect of the yeast mutant. Expression of the plant PEX11a or PEX11b proteins in *pex11*Δ cells partially complemented the oleate utilization defect, whereas no complementation could be observed with the human PEX11γ (Figure 1A).

We visualized peroxisomes through expression of the marker protein mCherry-px and counted red fluorescent dots in individual cells after induction of peroxisome proliferation. While most wild-type cells contained 4–9 peroxisomes, a significant fraction (>20% of the cells) contained 10 peroxisomes or more. In contrast, *pex11*Δ cells rarely contained more than seven peroxisomes (Figures 1B and S1A). Expression of the heterologous proteins *Hs*Pex11α, *Hs*Pex11β or *At*Pex11a-d in *pex11*Δ cells did not substantially alter the number of peroxisomes per cell and in most cases 20% of the cells contained one or two peroxisomes. However, upon expression of *Hs*Pex11β or *At*Pex11e more than 15% of the cells contained more than seven peroxisomes and only few cells contained one or two peroxisomes (<10%).

### EGFP-tagged Pex11 proteins localize to peroxisomes

**Figure 2:**
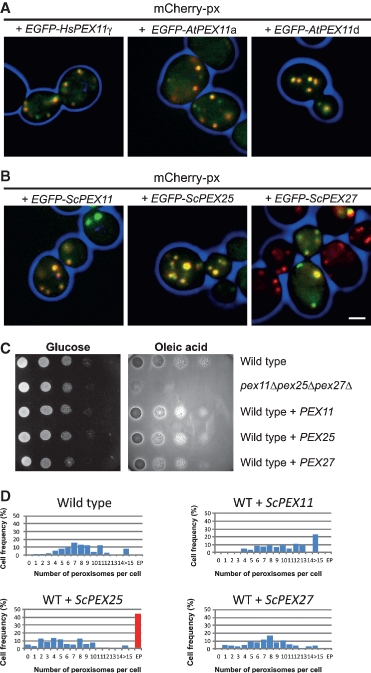
Localization of EGFP-tagged Pex11 proteins in yeast cells A) Wild-type yeast cells expressing the fluorescent peroxisomal marker protein mCherry-px (red channel) and transformed with plasmids expressing the EGFP-tagged version of *Hs*Pex11γ, *At*Pex11a or *At*Pex11d (green channel) under the control of the *GAL1* promoter were incubated overnight in medium containing glucose, and then in galactose for 4 h. B) Wild-type yeast cells expressing mCherry-px (red channel) and EGFP-tagged *Sc*Pex11p, *Sc*Pex25p or *Sc*Pex27p (green channel) controlled by the GPD promoter were incubated with oleic acid-containing medium for 16 h. Images represent single *Z*-layers. Bar: 2 mm. C) Wild-type yeast cells expressing mCherry-px and in addition *GPD*-controlled ScPex11p, ScPex25p or ScPex27p were spotted onto agar plates, and oleate utilization was monitored as described in Figure 1B. D) Wild-type yeast cells expressing mCherry-px (red channel) and *Sc*Pex11p, *Sc*Pex25p or *Sc*Pex27p were incubated in oleic acid-containing medium for 16 h. For each strain the fluorescent dots were counted in 100 non-budding cells. The histograms illustrate the frequency of cells with a distinct number of peroxisomes. The red bars indicate the frequency of cells containing elongated peroxisomes (EP). Note that the fraction of cells with elongated peroxisomal structures (red bar) is not included in the histograms presenting the peroxisome counts (blue bars).

**Figure 3:**
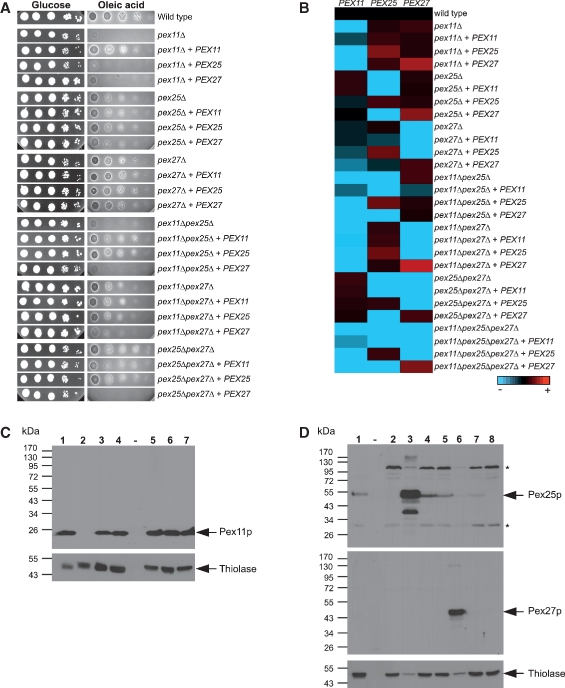
Overexpression of *Sc*Pex11p, *Sc*Pex25p or *Sc*Pex27p in yeast cells affects the function of peroxisomes A) Yeast mutant cells expressing Pex11p, Pex25p or Pex27p as indicated were grown to logarithmic phase in medium containing glucose. Then, 10-fold serial dilutions were spotted onto agar plates and oleate utilization was monitored by means of halo formation in the agar. B) Quantitative real-time PCRs were performed with mRNAs obtained from yeast cells lacking one, two or all three *PEX11* genes and from mutants expressing *PEX11*, *PEX25* or *PEX27* from plasmids as indicated. Cells were incubated in oleic acid-containing medium for 16 h. The levels were compared to mRNA levels in wild-type cells. Black indicates wild-type mRNA levels; a decrease in mRNA level is indicated by varying intensities of blue color; intensities of red color correspond to an increase in mRNA levels. The colored bar represents mRNA levels between 1/10 and 10-fold of wild-type levels. C) Western blot analysis of protein levels in wild-type cells (lane 1), *pex11*Δ (lane 2), *pex25*Δ (lane 3), *pex27*Δ cells (lane 4), and in *pex11*Δ*pex25*Δ cells (lane 5), *pex25*Δ*pex27*Δ cells (lane 6) and *pex11*Δ*pex25*Δ*pex27*Δ cells (lane 7) expressing *GPD*-promoter-controlled *Sc*Pex11p. Anti-Pex11p antibodies were used to visualize the Pex11 protein and thiolase was analyzed as loading control. D) Western blot analysis of protein levels in wild-type cells (lane 1), *pex11*Δ*pex25*Δ*pex27*Δ cells (lane 2), *pex11*Δ*pex25*Δ cells expressing *Sc*Pex25p from the *GPD* promoter (lane 3) or the *PEX25* promoter (lane 4), *pex11*Δ*pex25*Δ*pex27*Δ cells expressing *Sc*Pex25p from the *PEX25* promoter (lane 5), *pex11*Δ*pex27*Δ cells expressing *Sc*Pex27p either from the *GPD* promoter (lane 6) or the *PEX27* promoter (lane 7), and in *pex11*Δ*pex25*Δ*pex27*Δ cells expressing *Sc*Pex27p from the *PEX27* promoter (lane 8). Anti-Pex25p or anti-Pex27p antibodies were used to visualize the respective proteins and thiolase was used as the loading control. Less amount of protein was loaded in lanes 3 and 6 to avoid interference of strong signals with neighboring lanes. Asterisks indicate non-specific protein bands recognized by the antibody.

Considering the functional differences between the various heterologous Pex11 proteins we sought to analyze whether these also localize to peroxisomes in yeast cells. Interestingly, regardless of their ability to complement the oleic acid utilization phenotype, all enhanced green fluorescent protein (EGFP)-tagged Pex11 proteins localized to peroxisomes in wild-type cells (Figure 2A). Similarly, N-terminally EGFP-tagged Pex11p, Pex25p and Pex27p colocalized with mCherry-px (Figure 2B). These observations illustrate that even highly expressed, all Pex11 proteins localize to peroxisomes (see Figure 3B–D).

### Pex27p negatively affects peroxisomal function

**Figure 4:**
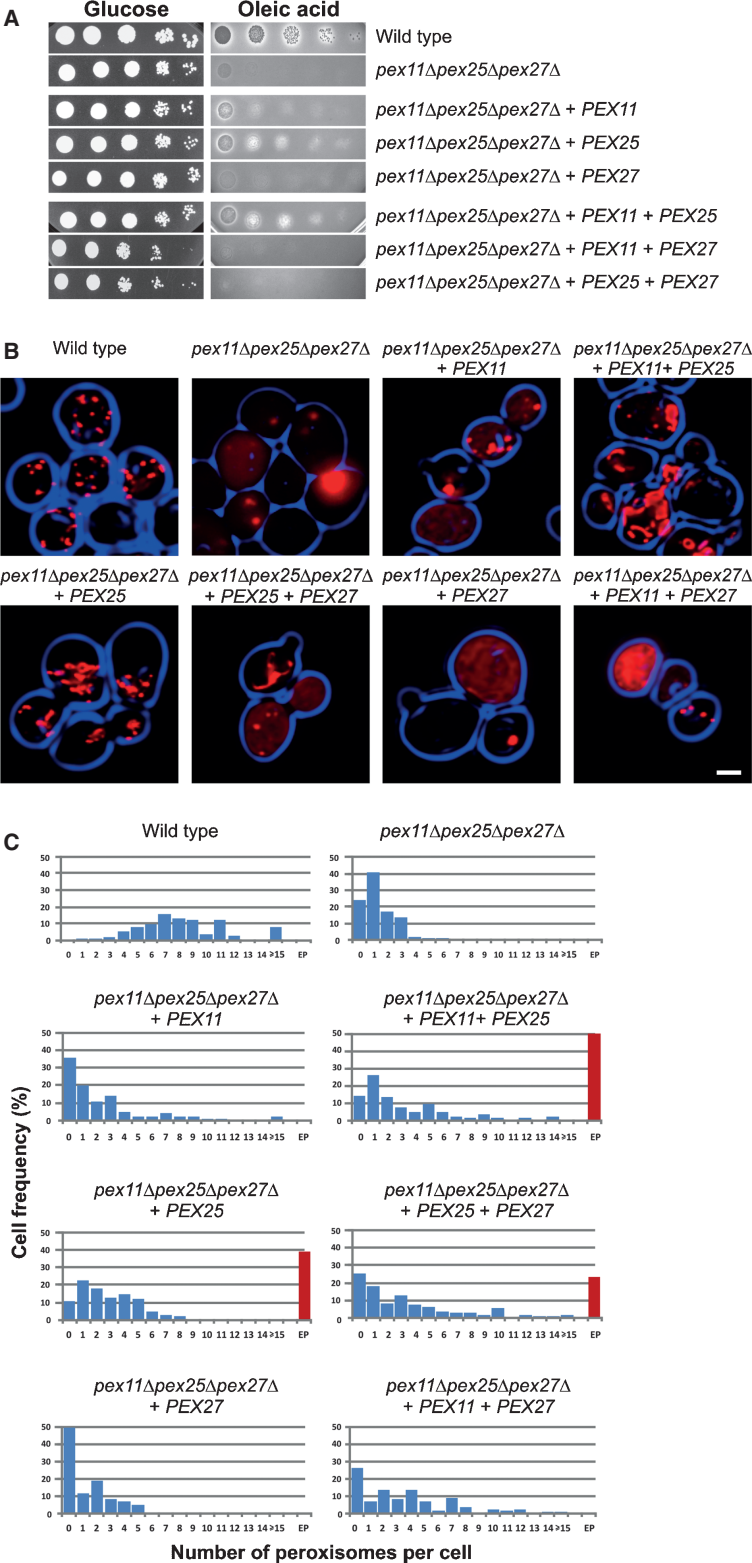
Each member of the Pex11p family differently affects function and number of peroxisomes A) Yeast mutants lacking *PEX11*, *PEX25* and *PEX27* (*pex11*Δ*pex25*Δ*pex27*Δ) and transformed with plasmids expressing Pex11p, Pex25p or Pex27p, Pex11p and Pex25p, Pex11p and Pex27p or Pex25p and Pex27p were tested for peroxisomal function through the halo assay. B) Cells described in (A) were incubated in medium containing oleic acid as the sole carbon source for 16 h. Peroxisomes were visualized by fluorescence microscopy (mCherry-px; red channel). Images represent deconvolved projected *Z*-stacks. Bar: 4 µm. C) Quantitative distribution of peroxisomes in cells described in (A) incubated for 16 h in oleic acid-containing medium. For each strain, fluorescent dots (mCherry-px) were counted in 100 non-budding cells. The histograms illustrate the frequency of cells with a distinct number of peroxisomes. The red bars indicate the frequency of cells containing elongated peroxisomes (EP). Note that the fraction of cells with elongated peroxisomal structures is not included in the histograms presenting the peroxisome counts (blue bars).

We examined whether the three members of the yeast Pex11 family could mutually compensate for each other's function. We employed a variety of yeast mutants deleted for *PEX11*, *PEX25, PEX27* or combinations thereof, tested their ability to utilize oleate and counted peroxisomes in the cells. In addition, to study the effects of individual Pex11 family members, we expressed one of the three Pex11 proteins in these mutant cells (Figure 3A). Generally, cells lacking Pex11p did not utilize oleate, but the concomitant absence of Pex27p restored the ability of *pex11*Δ cells to utilize oleate (*pex11*Δ*pex27*Δ and *pex11*Δ*pex27*Δ + *PEX25*). These results point for the first time to a negative effect of *Sc*Pex27p on peroxisomal function. In accordance with such a negative effect, overexpression of *Sc*Pex27p in *pex25*Δ*pex27*Δ cells resulted in the inability to utilize oleate (Figure 3A) and in *pex11*Δ*pex25*Δ*pex27*Δ cells significantly increased the fraction of cells without peroxisomes (50%; Figure 4). The negative action of *Sc*Pex27p could be based on competition with *Sc*Pex25p. In agreement, overexpression of *Sc*Pex27p in *pex11*Δ*pex27*Δ cells resulted in the reduced ability to utilize oleate (Figure 3A).

### Pex25p catalyzes membrane elongation

Furthermore, we asked whether Pex11p, Pex25p and Pex27p exerted different functions when expressed in *pex11*Δ*pex25*Δ*pex27*Δ cells (Figure 4). As reported previously (18,19), the expression of Pex11p in these cells only partially restored oleate utilization and resulted in a slight increase in the fraction of cells (33%) lacking peroxisomes. In contrast to the negative effect of Pex27p, when Pex25p was overexpressed, the cells could utilize oleate and produced more peroxisomes per cell leaving only a small portion (11%) of cells without peroxisomes. Strikingly, the expression of Pex25p from a plasmid was always associated with the appearance of elongated organelles (Figure 4B,C) reminiscent of juxtaposed elongated peroxisomes (JEPs) previously described in human cells upon ectopic expression of several members of the Pex11 protein family (3). The occurrence of these structures was enhanced upon concomitant expression of *PEX11* and *PEX25* from plasmids (Figure 4C). In contrast, when Pex27p was simultaneously expressed with either Pex11p or Pex25p, the negative effect of Pex27p on peroxisome function prevailed. Overexpression of *Sc*Pex25p from a plasmid compensated for the oleate utilization defect of *pex11*Δ*pex25*Δ mutants (Figure 3A). These combined data suggest that *Sc*Pex25p plays a key role in peroxisome biogenesis.

### Pex11p, Pex25p and Pex27p are expressed independently

The various effects of Pex11p, Pex25p and Pex27p on the number of peroxisomes and the formation of elongated peroxisomes could be the result of functional interdependence of the three proteins. Alternatively, this could reflect a mutual influence on gene expression. To distinguish between these possibilities, we analyzed gene expression and protein levels in yeast cells transformed with different plasmids (Figure 3B–D). Deletion or ectopic expression of any of the Pex11 protein-encoding genes had no significant influence on the mRNA levels of the others ruling out a mutual effect on transcription. The mRNA and protein of genomically expressed *PEX11* reached higher levels compared to plasmid-born expression controlled by the glyceraldehyde-3-phosphate dehydrogenase (*GPD*) promoter. This observation reflects the abundant production of Pex11p upon oleate induction. In contrast, *GPD*-driven expression of *PEX25* and *PEX27* was drastically increased as compared to their genomic expression levels (Figure 3B–D) and the proteins localized to peroxisomes (Figure 2B). These results indicated that expression levels of the three proteins are independent of each other, but do not exclude a mutual control through post-translational modifications.

### ScPex11p and ScPex25p alter the number of peroxisomes in wild-type cells

To investigate their diverse functions we expressed the Pex11 proteins from plasmids in wild-type cells. The ability of these cells to utilize oleic acid was not drastically altered compared to wild-type cells (Figure 2C). However, additional expression of Pex11p resulted in a higher number of peroxisomes per cell, and overexpression of Pex25p correlated with the appearance of elongated peroxisomal structures (Figure 2D). In contrast, cells overexpressing Pex27p displayed almost wild-type levels of peroxisomes. A plausible explanation for the lack of effect due to Pex27p overexpression is that its function might only be required under exceptional circumstances.

### Pex25p is a key player in *de novo* formation of peroxisomes

**Figure 5:**
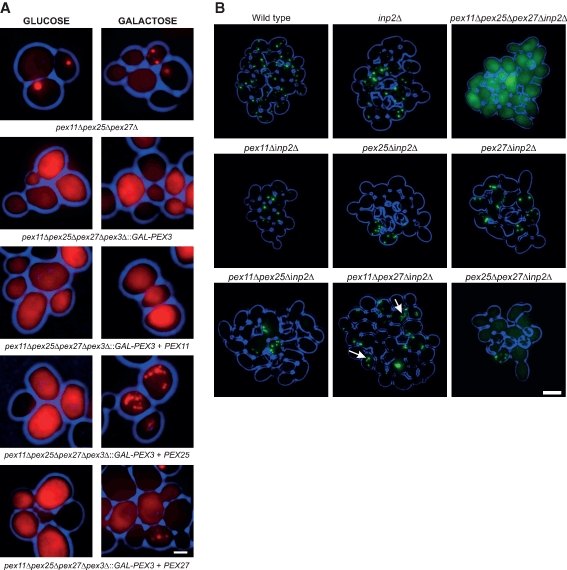
*De novo* formation of peroxisomes requires Pex25p A) Mutant cells lacking *PEX11*, *PEX25* and *PEX27*, expressing *PEX3* under the control of the *GAL* promoter (*pex11*Δ*pex25*Δ*pex27*Δ*pex3*Δ::*GAL*-*PEX3*) and transformed with plasmids expressing Pex11p, Pex25p or Pex27p were grown in the presence of 2% glucose with or without a short period of exposure to 2% galactose 8 h prior to microscopic inspection. Peroxisomes were visualized by fluorescence microscopy (mCherry-px; red channel). Bar: 2 µm. B) Microscopic analysis of yeast cells lacking *INP2* and one, two or three members of the *PEX11* family and expressing GFP-px under the control of the *GAL* promoter (green channel). The corresponding DNA sequence was integrated into the *INP2* locus. After growth on glucose for 16 h, cells were shifted to medium containing 1% raffinose and 2% galactose for 6 h and thinly seeded on microscope slides covered with agarose containing the same medium. After 10 h, colonies originating from single cells were inspected for the distribution of green fluorescence emitted from GFP-px. Arrows point to elongated peroxisomes. Images in (A) and (B) represent projected *Z*-stacks. Bar: 5 µm.

Yeast cells lacking the protein Pex3p are devoid of peroxisomes but reintroduction of a functional Pex3 protein leads to full peroxisomal recovery (27,28). While mutant cells lacking all three Pex11-related proteins (pex11Δpex25Δpex27Δ) contained up to three peroxisomes (see Figure 4), the additional lack of Pex3p led, as expected, to the complete absence of peroxisomes (Figure 5A). To analyze the effects of Pex11 family members on peroxisome biogenesis, we established an experimental set up allowing the reintroduction of Pex3p upon change of carbon source. We replaced the genuine *PEX3* promoter with a galactose-inducible *GAL* promoter, whose expression is turned off in the presence of glucose. The cells (*pex11*Δ*pex25*Δ*pex27*Δ*pex3*Δ::*GAL*-*PEX3*) remained void of peroxisomes even after activation of Pex3p synthesis by growth on galactose (Figure 5A). This result demonstrated that at least one of the three Pex11 family members is required for the regeneration of peroxisomes after their complete loss. Using simultaneous expression of *PEX3* via growth of cells on galactose and either *PEX11*, *PEX25* or *PEX27* from plasmids, we asked which one of the three proteins is required for *de novo* formation of peroxisomes (Figure 5A). Wild-type levels of peroxisomes were only restored in mutant cells when *PEX25* was expressed together with *PEX3*. Moreover, elongated peroxisomes were visible in the course of peroxisome generation. The expression of *PEX27* in conjunction with *PEX3* allowed the formation of few peroxisomes in a limited number of cells (<10%). Indicating that Pex11p has no function in *de novo* formation of peroxisomes, the combined expression of *PEX3* and *PEX11* did not lead to the formation of peroxisomes. Taken together, these data suggest that *Sc*Pex25p is an essential factor for *de novo* biogenesis of peroxisomes and that *Sc*Pex27p has the capacity to partially substitute for the role of *Sc*Pex25p.

If Pex25p is indeed required for *de novo* biogenesis, this protein should be essential to regain peroxisomes in inheritance mutants. Therefore, we employed *inp2*Δ mutant cells, in which peroxisomes are retained in the mother cells during cell division (29). However, peroxisomes can slowly form in *inp2*Δ cells. While daughter cells are temporarily devoid of peroxisomes, after a full generation they seem to assemble these organelles *de novo*(10). Consequently, in a colony arising from a single budding *inp2*Δ cell, approximately half of the cells are expected to contain peroxisomes. If *de novo* biogenesis is impaired then most cells will lack peroxisomes. To visualize peroxisomes the coding sequence for the marker protein GFP-px was integrated into the genome replacing *INP2* in cells additionally deleted for different combinations of *PEX11*, *PEX25* and *PEX27*. The cells were thinly seeded onto agarose containing growth medium and allowed to form microcolonies prior to microscopic analysis (Figure 5B). As expected, in *inp2*Δ colonies half of the cells contained green fluorescent dots indicating the presence of peroxisomes. The *inp2*Δ mutants additionally lacking all three Pex11 proteins presented a cytosolic green staining without punctae, indicating the absence of peroxisomes. Few peroxisomes were visualized in most *pex11*Δ*pex25*Δ*pex27*Δ mutant cells (see Figure 4). The additional loss of *INP2* would require *de novo* formation of peroxisomes in the daughter cells which obviously did not occur in the absence of the three Pex11 family members. In colonies originating from cells lacking *INP2* and *PEX11* (*pex11*Δ*inp2*Δ), half of the cells contained peroxisomes, and these cells contained a smaller number of peroxisomes. These results suggest that Pex11p is not involved in *de novo* biogenesis but rather functions in determining the number of peroxisomes present in each peroxisome-containing cell. In the absence of Pex27p (*inp2*Δ*pex27*Δ), colonies were indistinguishable from those originating from *inp2*Δ mutant cells (Figure 5B). Similarly, when only Pex25p was expressed (*pex11*Δ*pex27*Δ*inp2*Δ), half of the cells contained peroxisomes. Moreover, elongated peroxisomes were visible in a number of these cells (Figure 5B). In colonies originating from *pex25*Δ*inp2*Δ cells, only 10% of cells contained peroxisomes, and the majority of cells showed a diffuse cytosolic fluorescence. These inheritance assays demonstrated that Pex25p plays an essential role in the *de novo* formation of peroxisomes. Its function can at least be partially substituted by Pex27p, because in colonies originating from *pex11*Δ*pex25*Δ*inp2*Δ single cells, about 10% of cells enclosed peroxisomes. Further supporting this notion, less than 5% of *pex25*Δ*pex27*Δ*inp2*Δ mutants contained peroxisomes, and several colonies originating from these mutants were observed with cells completely devoid of peroxisomes. Notably, numerous peroxisomes could be observed in the very few peroxisome-containing cells found in *pex25*Δ*pex27*Δ*inp2*Δ mutant colonies. Again this finding supports the central role for Pex11p in the regulation of the number of peroxisomes per cell.

## Discussion

To determine the overall function of Pex11 proteins, we explored the potential of heterologous Pex11 proteins to complement the *pex11*Δ phenotype of *S. cerevisiae* cells (Figure 1). The *Sc*Pex11 protein is more closely related to the heterologous Pex11 proteins than to the two other family members present in *S. cerevisiae*, Pex25p and Pex27p (30). Expression of the heterologous Pex11 proteins did not significantly change the number of peroxisomes in *pex11*Δ cells. While they all localized to peroxisomes, only five of the eight proteins tested allowed the cells to consume oleic acid (Figures 1A and 2A). Noteworthy, among the five proteins complementing the oleate utilization phenotype, all but *Hs*PEX11β contain at their C-terminus a −KXKXX motif known as ER-retrieval signal (31). This motif may already indicate a connection of these peroxisomal proteins with the ER.

The three members of the Pex11 family in *S. cerevisiae* were originally identified as factors controlling peroxisome number and function (16,18,19,32). However, their individual contribution remained unknown. To discern their particular roles we expressed these proteins in single, double and triple mutants alone or in combinations, analyzed the ability of transformed cells to utilize oleate and evaluated the number of peroxisomes per cell (Figures 3A and 4). Our results provide evidence that each member of the Pex11 family holds a different function in the control of peroxisome number and metabolic activity. This suggests that in *S. cerevisiae*, the delicate balance between Pex11p, Pex25p and Pex27p ensures a variable number of peroxisomes and guarantees that each cell is furnished with adequate peroxisomal metabolism.

Several characteristics suggest a role for *Sc*Pex11p and close heterologous relatives in membrane remodeling. Its conserved localization (Figure 2) (3,23,30), its abundance (16) and the lack of a transmembrane domain but presence of an amphipathic helix (15) might allow for exclusion or specific association of proteins or metabolites at the peroxisomal membrane. Amphipathic helices are thought to sense membrane curvature or to participate in membrane remodeling (33). Thus, Pex11p might act as a sensor to determine the ability of the membrane to proliferate. Alternatively, a continuous gradient of Pex11p might allow for membrane protrusion. In both cases, accumulation of Pex11p at specific membrane sites with a precise form or lipid composition might influence the peroxisomal metabolism, a behavior that seems to have been conserved throughout evolution.

In the mere absence of Pex11p, cells are unable to utilize fatty acids. Interestingly, the additional lack of Pex27p allowed the cells to regain peroxisomal function (Figure 3A), suggesting a negative or competitive role for Pex27p. Consistent with this notion, the reintroduction of Pex27p into *pex11*Δ*pex27*Δ cells and *pex25*Δ*pex27*Δ cells reduced their ability to utilize oleate (Figure 3A). While overexpression of Pex25p in *pex11*Δ*pex25*Δ*pex27*Δ cells led to the occurrence of elongated peroxisomal structures, the concomitant expression of Pex25p and Pex27p in these cells reduced the frequency of elongated peroxisomes (Figure 4B,C). The presence of functional peroxisomes in *pex11*Δ*pex27*Δ cells and the occurrence of fewer cells with elongated peroxisomes in *pex11*Δ*pex25*Δ*pex27*Δ mutants expressing Pex25p and Pex27p from plasmids are observations in agreement with a model in which Pex27p competes with Pex25p during the process of proliferation. The finding that Pex27p can partially substitute for Pex25p in *de novo* formation of peroxisomes strongly supports a competition between these two proteins (Figure 5B), suggesting a similar role and a similar localization for both (18). That this process is slow in the absence of Pex25p could be because of the fact that endogenous Pex27p is only present in small amount in wild-type yeast cells (18). However, in contrast to Pex11p and Pex25p, overexpression of Pex27p does not lead to functional peroxisomes in cells lacking all three proteins (Figure 4A). In wild-type cells, overexpression of Pex27p showed only moderate influence on peroxisomal number. An explanation could be that Pex27p is only active when the balance between Pex11p and Pex25p is perturbed which could endanger the propagation of peroxisomes.

The negative effect exerted by Pex27p on peroxisomal function most likely takes place at the peroxisomal membrane. In wild-type cells, the presence of Pex25p or Pex27p at the peroxisomal membrane could locally alter the lipid-to-protein ratio, thereby enhancing the association of Pex11p with the membrane at this site. This in turn would result in Pex11p accumulation, membrane remodeling and proliferation at this exact site. The property of Pex11p to oligomerize (20) might support a co-operative association with the peroxisomal membrane, which, in turn, could explain its function in proliferating peroxisomes already present in the cell.

In the absence of Pex11p and Pex27p, Pex25p is sufficient to provide the cells with functional peroxisomes. The occurrence of elongated peroxisomes (Figure 5B), strongly increased upon ectopic expression of Pex25p (Figure 4B), suggests that this protein triggers membrane elongation, a step essential to prime peroxisome proliferation. Pex27p might compete with Pex25p in the process of membrane association or at the level of protein interaction, e.g. with Pex11p. However, as there is no evidence for heteromeric interactions between Pex11 family members, the interplay between these proteins might rather rely on the interaction of each individual protein with lipids of the same (peroxisomal) membrane. We propose that the interaction between each member of the Pex11 protein family and the peroxisomal membrane has been conserved throughout evolution. This hypothesis fits the observation that heterologous Pex11 proteins localize to peroxisomes and compensate for the loss of peroxisomal function to various degrees (Figure 2) (3,23,30).

Growth and division of existing peroxisomes and *de novo* formation from the ER constitute the peroxisome biogenesis (10,34). The protein Pex3p was previously described as an early peroxisome biogenesis factor and it was shown to be the initiating factor for peroxisome biogenesis from the ER (9,35). While proliferation from existing peroxisomes could take place in the absence of the Pex11-family members, after loss of peroxisomes, Pex25p was required to generate wild-type levels of peroxisomes. Hence, we demonstrate that Pex25p acts in intimate co-operation with Pex3p and that both are equally required for *de novo* formation. Similar results were obtained in the yeast *Hansenula polymorpha*(36).

**Figure 6:**
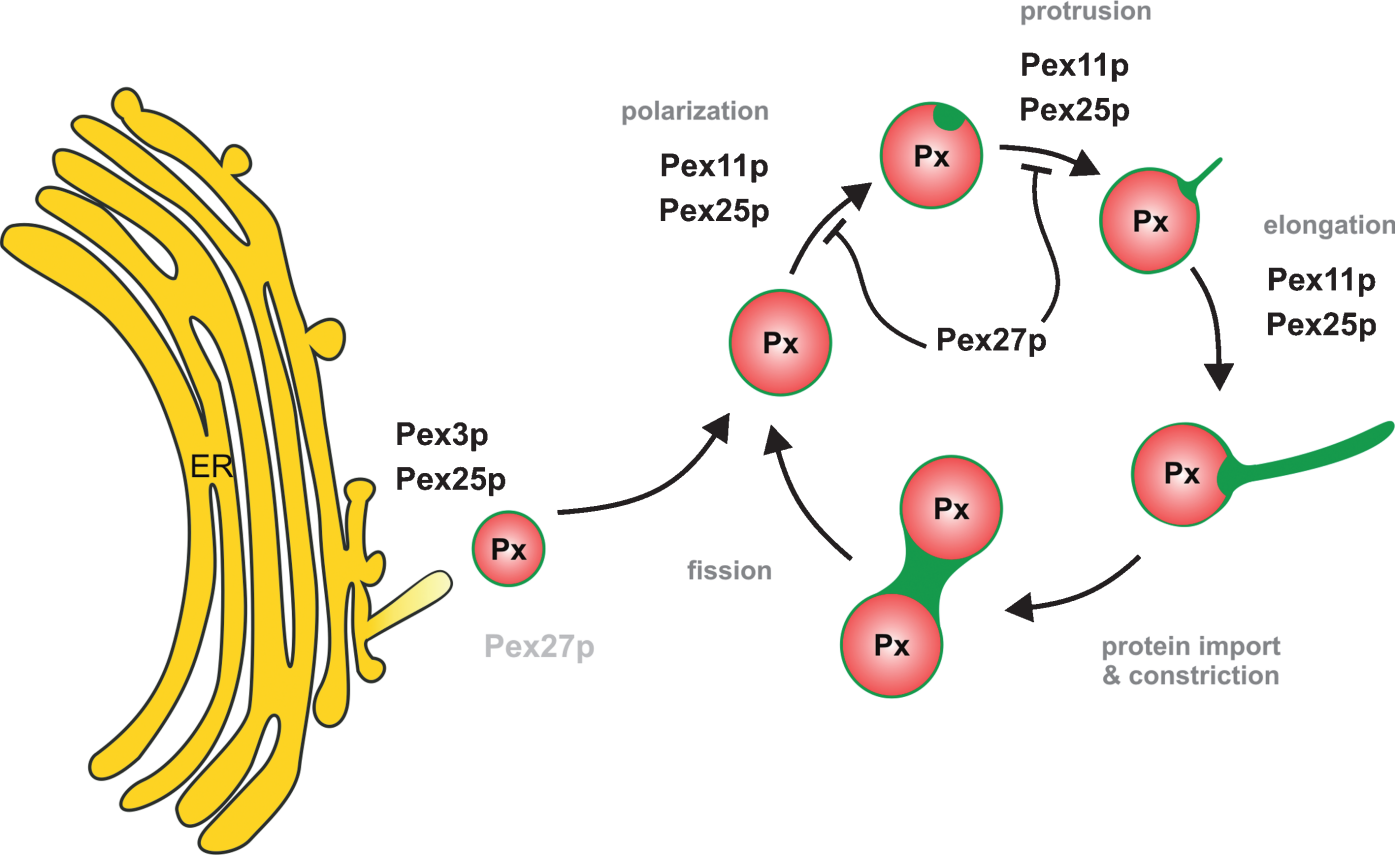
A model for the function of Pex11p, Pex25p and Pex27p in peroxisome biogenesis Consistent with the previous models on Pex11 protein function (3,14,15), *Sc*Pex11p serves as a sensor to determine the ability of the peroxisomal membrane to proliferate. Supported by its ability to co-operatively associate, *Sc*Pex11p accumulates at specific membrane sites, which leads to membrane elongation and protrusion. *Sc*Pex25p might catalyze this priming event for peroxisomal membrane elongation preparing the membrane for association and accumulation of *Sc*Pex11p. And *Sc*Pex25p is also able to provide yeast cells with functional peroxisomes on its own. These particular functions of *Sc*Pex25p in proliferation of existing peroxisomes are inhibited or competed by *Sc*Pex27p. In addition, *Sc*Pex25p plays together with Pex3p an important role in initiating the *de novo* formation of peroxisomes. Here, *Sc*Pex27p most likely acts as a structural component (indicated in gray) which partially substitutes for the function of *Sc*Pex25p.

We present a model (Figure 6), in which each one of the yeast Pex11 proteins holds an individual function in the formation of peroxisomes. In conclusion, (i) we demonstrate that Pex25p participates in membrane elongation of existing peroxisomes and in the initiation of *de novo* biogenesis from the ER, (ii) we provide evidence that Pex27p exerts an inhibitory or competitive function and (iii) we show that Pex11p only promotes the proliferation of peroxisomes already present in the cell.

## Materials and Methods

### Plasmids

The pENTR4-*PEX11* plasmids (3) were recombined (Gateway, Invitrogen) with plasmid pRS413-*GPD*prom-*ccdB* (pCB623) to generate plasmids expressing the *PEX11* genes under the control of the *GPD* promoter (for plasmids used in this study see Table S1). In addition, plasmids #1 (*ScPEX11*), #25 (*ScPEX25*) and #74 (*ScPEX27*) were recombined with plasmid pRS415-*GPD*prom-*ccdB* (pCB826). To generate plasmids expressing N-terminally EGFP-tagged versions of the Pex11 proteins, plasmids #1 (*Sc*PEX11), #25 (*Sc*PEX25), #74 (*Sc*PEX27), #217 (*Hs*PEX11γ), #102 (*At*PEX11A) and #105 (*At*PEX11D) were recombined with plasmids pRS413-GALprom-EGFP-ccdB (pCB630) or pRS413-GPDprom-EGFP-ccdB (pCB631). The promoter and coding sequences of PEX25 and PEX27 were amplified by PCR using genomic yeast DNA as template and the primer pairs Pex25y-1/Pex25y-2 and Pex27y-1/Pex27y-2, respectively. The DNA fragments obtained were cloned into pGEM-T (Stratagene) and then into pRS313 (*Xba*I/*Not*I) to produce plasmids 1087 and 1088, respectively. mCherry-px was amplified by PCR using pCB314 as a template and primer pair CB111/CB112 and cloned into pCB441 (*Bam*HI/*Hin*dIII) to produce pCB367. The primer pair H911/H912 and plasmid pCB761 were used to amplify the *ADH1* promoter. The PCR fragment was cloned into YEplac195 (37; *Sac*I/*Xba*I) to obtain pCB619. Then, the mCherry-px coding sequence was amplified using primer pair CB293/CB112 (template pCB314) and subcloned into pCB619 resulting in plasmid pCB741. A PCR fragment coding for *GAL*-Sprom-*yeGFP*-px was produced using the primer pair CB344/CB345 and template pCB516 and introduced into plasmid pCB447 (*Sac*I) to obtain plasmid pCB840.

### Strains, media and growth conditions

*Escherichia coli* strains DH5α or DB3.1 (DEST vectors) were used for cloning. The *PEX11* gene was deleted via homologous recombination in CB80 (38) using a PCR fragment amplified from plasmid pFA6-KanMX4 (Euroscarf) with primer pair Pex11-y-24 and Pex11-y-25 giving rise to strains CB369 and CB370 (for yeast strains used in this study see Table S3). Strains CB371, CB372, CB374, CB375, CB376, CB417 and CB419 were obtained by crossing. Plasmid pCB367 was linearized (EcoRV) and integrated into the URA3 locus of the yeast genomes of CB80, CB369 and CB419 to obtain the strains CB515, CB516, CB545, respectively. In strain CB419, the *PEX3* promoter was replaced by the *GAL*-S promoter using plasmid pCB514 and primers CB206/CB207 to obtain strain CB547. The *LEU2* locus in CB80 was deleted using a PCR fragment (primers CB352/CB353) derived from plasmid pCB840 resulting in strain CB537. To obtain strains CB541, CB542, CB543, CB544, CB532, CB533 and CB535, CB536 *INP2* was deleted using a PCR fragment amplified from plasmid pCB840 with primer pair CB346/CB347 in strains CB80, CB369, CB371, CB372, CB374, CB376, CB417 and CB419, respectively. Yeast strains were grown to optical density at 600nm (OD_600_) = 1 in glucose medium (0.3% glucose, 0.67% yeast nitrogen base without amino acids (YNB), 0.1% yeast extract, supplemented with amino acids and bases as required, pH 6 with KOH). Then, 5xYNO medium (3.35% yeast nitrogen base without amino acids, supplemented with amino acids and bases as required, pH 6 with KOH, 0.25% Tween-80, 0.5% oleic acid) was added and cells were grown for 16 h. For halo assays, 10-fold serial dilutions of logarithmically growing cultures (OD_600_ = 1) were spotted onto yeast-extract peptone dextrose (YPD) plates (2% glucose, 1% yeast extract, 2% peptone, 3% agar) or oleic acid plates (0.67% YNB, 0.1% yeast extract, 0.125% oleic acid, 0.5% Tween-80, 0.5% KH_2_PO_4_, pH 6 with K_2_HPO_4_, supplemented with amino acids and bases as required, 3% agar). Cells expressing *PEX3* under the *GAL*-S promoter were incubated in medium containing 2% glucose with or without a short incubation period in medium containing 2% galactose 8 h prior to microscopic inspection. Cells expressing GFP-px from the *GAL*-S promoter after integration into the *INP2* locus were grown in a synthetic complete medium (SC) with 2% glucose, transferred to SC with 2% galactose/1% raffinose for overnight growth, thinly seeded onto agarose pads containing SC with 2% galactose/1% raffinose and incubated at 30°C for 10 h. Cells expressing heterologous EGFP-tagged Pex11 proteins from the GAL1 promoter on plasmids were grown overnight in glucose medium (0.3% glucose, 0.67% yeast nitrogen base without amino acids, 0.1% yeast extract, supplemented with amino acids and bases as required, pH 6 with KOH), transferred to glucose-free medium containing 1% galactose as carbon source and grown for 4 h prior to fluorescence imaging.

### Quantitative real-time PCR

Total RNAs were isolated from yeast using standard procedures. cDNAs were synthesized using Oligo(dT)_18_ primer and RevertAid™ Premium Reverse Transcriptase (Fermentas). PCRs were performed in triplicates with RedTaq2.0xMasterMix (1.5 mm MgCl_2_; VWR), SYBR Green (Invitrogen), fluorescein isothiocyanate (FITC) (BioRad), deoxyribonucleotide triphosphates (dNTPs) (Roche) and primer pairs Pex11-y-50/51, Pex25-y-12/13 or Pex27-y-15/16 in 96-well plates using a BioRad ICycler. ΔΔC_T_ values were calculated and the TreeView software was used to illustrate the results.

### Antibodies

Anti-Pex11p and anti-Pex25p antibodies were generated in rabbits using the following peptides Pex11: KAKSQSQGDEHEDHKKVLG and Pex25: GASYQDAQDDNTHPHSSDA (Davids Biotechnologie GmbH). Horseradish peroxidase-conjugated antibodies sheep-anti-mouse and donkey-anti-rabbit (GE Healthcare) were purchased. Rabbit anti-thiolase antibodies and rabbit anti-Pex27p antibodies were kindly supplied by Wolf Kunau (Bochum, Germany) and Richard Rachubinski (Edmonton, Canada), respectively.

### Microscopy and statistical analysis

All images were acquired with a wide-field microscope (Olympus CellR Imaging Station) equipped with the following filter sets: BP510-550 excitation; LP590 emission for mCherry; BP457-487 excitation; BP503-538 emission for GFP. Images were processed using ImageJ (NIH). Stacks were projected along the *z*-axis (maximum intensity), and brightness and contrast were adjusted for each channel. Transmission images were acquired, colored in blue and brightness and contrast were adjusted to display the borders of each cell. Images were deconvolved with the qmle algorithm using an experimentally derived PSF and the software Huygens Professional when indicated. The figures were composed in CorelDrawX4. For statistical analysis, images were acquired as described above (Olympus, CellR) and red dots (mCherry-px) were manually counted through the whole image stack for at least 100 randomly chosen cells. Only in cells with red fluorescence, either cytosolic or punctate, and without elongated structures, peroxisomes were counted. The histograms were generated in Microsoft Office Excel.

## Supporting Information

**Figure S1: Overexpression of Pex11 proteins in yeast mutantcells affects peroxisome number.** A) Yeast cells lacking *PEX11* were transformed with plasmids expressing Pex11 proteins from different organisms and grown on oleic acid-containing media for 16 h. Peroxisomes were visualized through mCherry-px (red channel). Images represent projected *Z*-stacks. Bar: 2 µm. B) Yeast mutant cells as indicated were transformed with plasmids expressing Pex11p, Pex25p or Pex27p and grown on oleic acid-containing media for 16 h. Peroxisomes were visualized through mCherry-px (red channel).Images represent projected *Z*-stacks. Bar: 2 µm.

**Table S1:** Plasmids used in this study

**Table S2:** Oligonucleotides used in this study

**Table S3:** Yeast strains used in this study
