# Supplementary figures and images for: A Subtle Interplay Between Three Pex11 Proteins Shapes De Novo Formation and Fission of Peroxisomes
Source: Traffic. 2011 Oct 20;13(1):157–67. doi: 10.1111/j.1600-0854.2011.01290.x (PMC3245845; doi:10.1111/j.1600-0854.2011.01290.x)

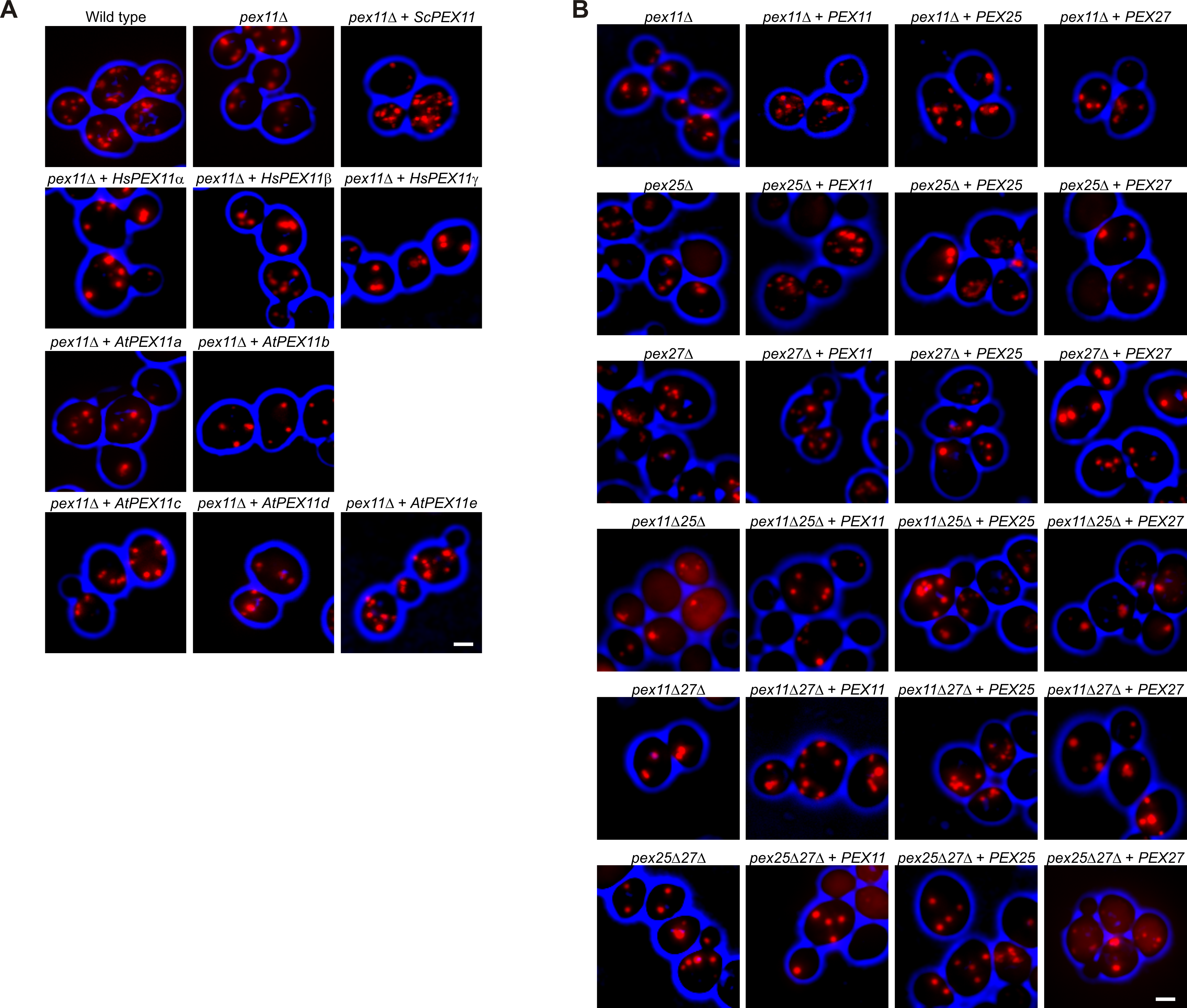

Supplement: Supplementary file 1 [file tra0013-0157-SD1.tif]
