# Supplementary material for: A Subtle Interplay Between Three Pex11 Proteins Shapes De Novo Formation and Fission of Peroxisomes
Source: Traffic. 2011 Oct 20;13(1):157–67. doi: 10.1111/j.1600-0854.2011.01290.x (PMC3245845; doi:10.1111/j.1600-0854.2011.01290.x)
Supplement: Supplementary file 2 [file tra0013-0157-SD2.pdf]

Table S1: Plasmids used in this study

| Plasmid | Description | Source |
|---|---|---|
| 1 | *ScPEX11* in pENTR4 | (3) |
| 25 | *ScPEX25* in pENTR4 | (3) |
| 74 | *ScPEX27* in pDONR-Zeo | (3) |
| 215 | *HsPEX11*$\alpha$ in pENTR4 | (3) |
| 297 | *HsPEX11*$\beta$ in pENTR4 | (3) |
| 217 | *HsPEX11*$\gamma$ in pENTR4 | (3) |
| 102 | *AtPEX11A* in pENTR4 | (3) |
| 104 | *AtPEX11B* in pENTR4 | (3) |
| 100 | *AtPEX11C* in pENTR4 | (3) |
| 105 | *AtPEX11D* in pENTR4 | (3) |
| 101 | *AtPEX11E* in pENTR4 | (3) |
| 98 | pRS413-*GPD*prom-*ScPEX11* | This study |
| 1026 | pRS413-*GPD*prom-*ScPEX25* | This study |
| 1031 | pRS413-*GPD*prom-*ScPEX27* | This study |
| 1039 | pRS413-*GPD*prom-*HsPEX11*$\alpha$ | This study |
| 1080 | pRS413-*GPD*prom-*HsPEX11*$\beta$ | This study |
| 1020 | pRS413-*GPD*prom-*HsPEX11*$\gamma$ | This study |
| 1023 | pRS413-*GPD*prom-*AtPEX11a* | This study |
| 1024 | pRS413-*GPD*prom-*AtPEX11b* | This study |
| 1021 | pRS413-*GPD*prom-*AtPEX11c* | This study |
| 1025 | pRS413-*GPD*prom-*AtPEX11d* | This study |
| 1022 | pRS413-*GPD*prom-*AtPEX11e* | This study |
| 1077 | pRS415-*GPD*prom-*ScPEX11* | This study |
| 1078 | pRS415-*GPD*prom-*ScPEX25* | This study |
| 1079 | pRS415-*GPD*prom-*ScPEX27* | This study |
| 1006 | pRS413-*GPD*prom-EGFP-*ScPEX11* | This study |
| 1047 | pRS413-*GPD*prom-EGFP-*ScPEX25* | This study |
| 1048 | pRS413-*GPD*prom-EGFP-*ScPEX27* | This study |
| 1063 | pRS413-*GAL*prom-EGFP-*ScPEX11* | This study |
| 1066 | pRS413-*GAL*prom-EGFP-*ScPEX25* | This study |
| 1068 | pRS413-*GAL*prom-EGFP-*ScPEX27* | This study |
| 1087 | pRS313-*ScPEX25* | This study |
| 1088 | pRS313-*ScPEX27* | This study |
| | | |
| pCB623 | pRS413-*GPD*prom-*ccdB* | Addgene |
| pCB630 | pRS413-*GAL*prom-EGFP-*ccdB* | Addgene |
| pCB631 | pRS413-*GPD*prom-EGFP-*ccdB* | Addgene |
| pCB572 | pRS313 | Addgene |
| pCB826 | pRS415-*GPD*prom-*ccdB* | Addgene |
| pCB314 | pmCherryGwB | Jenuwein |
| pJR233 | YEplac352-*MLS1*prom-GFP2-px | (38) |
| pCB367 | YEplac352-*MLS1*prom-*mCherry*-px | This study |
| pCB761 | p416-*ADH1*prom-mt-RFP | This study |
| pCB619 | YEplac195-*ADH1*prom | This study |
| pCB741 | YEplac195-*ADH1*prom-*mCherry*-px | This study |
| pCB370 | pFA6-*KanMX4* | Euroscarf |
| pCB447 | pFA6a-*hphNT1* | Euroscarf |
| pCB514 | pYM-N31-*GALS*prom-*natNT2* | Euroscarf |
| pCB516 | pYM-N33-*GALS*prom-*yeGFP-natNT2* | Euroscarf |
| pCB840 | pFA6a-*hphNT1-GALS*prom-*yeGFP*-px | This study |
