# Supplementary material for: A Subtle Interplay Between Three Pex11 Proteins Shapes De Novo Formation and Fission of Peroxisomes
Source: Traffic. 2011 Oct 20;13(1):157–67. doi: 10.1111/j.1600-0854.2011.01290.x (PMC3245845; doi:10.1111/j.1600-0854.2011.01290.x)
Supplement: Supplementary file 3 [file tra0013-0157-SD3.pdf]

Table S2: Oligonucleotides used in this study

Pex11-y-24	CTACTACTTCAAAGACTTCATCAAGTAATAGTATAATCAATCGTACGCTGCAGGTCGAC	Fwd-*ScPEX11* for deletion
Pex11-y-25	ATAAAGAAGGGTCGAATCAAACATAAGCGGAGAATAGCCATCGATGAATTCGAGCTCG	Rev-*ScPEX11* for deletion
Pex11-y-50	ATATCATCCCTCCGTGACGA	Fwd-*ScPEX11* for RT-PCR
Pex11-y-51	GCCTGCAAGTGGTTTAAAGG	Rev-*ScPEX11* for RT-PCR
Pex25-y-12	GGCTCACAATCGCCAATTA	Fwd-*ScPEX25* for RT-PCR
Pex25-y-13	GCATACGTTCCCTTTGGAGT	Rev-*ScPEX25* for RT-PCR
Pex27-y-15	CCCCACTTTAACGGACAGA	Fwd-*ScPEX27* for RT-PCR
Pex27-y-16	GCAGTAGCAACTTTTCCCTGA	Rev-*ScPEX27* for RT-PCR
Pex25-y-1	CGCCGCTATATGTGAAGGGC	Fwd-*ScPEX25* for cloning
Pex25-y-2	GCGTCAAAGTGTCAGAATAAAC	Rev-*ScPEX25* for cloning
Pex27-y-1	GGCAATAATATTAGGTATGTAGAA	Fwd-*ScPEX27* for cloning
Pex27-y-2	GGCGAGATGGAAATGTAGTCT	Rev-*ScPEX27* for cloning
CB111	TTAGGATCCGGTACCATGGTGAGCAAGGG	Fwd-*mCherry*-px in pCB441 (BamHI)
CB112	GCAAGCTTTTATAATTTGGACTCGATGTTTTTCTTGTACAGCTCGTCCATGCC	Rev-*mCherry*-px in pCB441 (HindIII)
H911	ATGCGAGCTCCCCCTCCGCGCTCTTTTCCG	Fwd-*ADH1*prom in YEplac195 (SacI)
H912	ATGCTCTAGAATCCGGGTGTATATGAGATAGTTGATTGTATGC	Rev-*ADH1*prom in YEplac195 (XbaI)
CB293	GCTCTAGAATGGTGAGCAAGGGCGAGGAGGATAAC	Fwd-*mCherry*-px in pCB619 (XbaI)
CB206	TACTAGTCATCGTAAAAGCAGAAGCACGAAACAAGGAGGCAAACCACTAAAAGGATGCGTACGCTGCAGGTCG	Fwd-*GAL*-Sprom-*Pex3* with pCB514
CB207	ATGAGTACCTTTCCTCGATGTCTCTGCAGAAGCGAACGTGATCTTTGATTTGGGGCCATCGATGAATTCTCTGTCG	Rev-*GAL*-Sprom-*Pex3* with pCB514
CB344	CCTGTTGTAATCGAGCTCGGAAGACTC	Fwd-*GAL*-Sprom in pCB447 (SacI)
CB345	CCTTGAGCTCTCATTATAATTTGGACTCGATGTTTTTTTTGTACAATTCATCCATACC	Rev-*yeGFP*-px in pCB447 (SacI)
CB346	CCTCACGCTTATTGCAACAAGTTTGTTTTTACTTACTTGTGAAACGTTTGTTG CTGAAGCTTCGTACGCTGCAGGTCGACGG	Fwd-*INP2* for deletion in pFA6
CB347	GTAATTAGTTATTTCAAAGTACATATTAAAATATATTATCATGAATCAGGATCTGATATCATCGATGAATTCGAGCTC	Rev-*INP2* for deletion in pFA6
CB352	CCTCAAAAAGATCCATGTATAATCTTCATTATTACAGCCCTCTTGACCCTGAAGCTTCGTACGCTGCAGGTCGACGG	Fwd-*LEU2* for deletion in pFA6
CB353	GTATGTAGATTGCGTATATAGTTTCGTCTACCCTATGAACATATTCCGGATCTGATATCATCGATGAATTCGAGCTC	Rev-*LEU2* for deletion in pFA6
