# Supplementary material for: A Subtle Interplay Between Three Pex11 Proteins Shapes De Novo Formation and Fission of Peroxisomes
Source: Traffic. 2011 Oct 20;13(1):157–67. doi: 10.1111/j.1600-0854.2011.01290.x (PMC3245845; doi:10.1111/j.1600-0854.2011.01290.x)
Supplement: Supplementary file 4 [file tra0013-0157-SD4.pdf]

Table S3: *S. cerevisiae* strains used in this study

Strain	Description	Mating	Source
CB80	Mat a his3-200, leu2-1, trp1-63, ura3-52		(38)
CB80	Mat α his3-200, leu2-1, trp1-63, ura3-52		(38)
CB515	Mat a his3-200, leu2-1, trp1-63, ura3::MLS1prom-mCherry-px		This study
CB369	Mat a his3-200, leu2-1, trp1-63, ura3-52, pex11::kanMX4		This study
CB370	Mat α his3-200, leu2-1, trp1-63, ura3-52, pex11::kanMX4		This study
CB516	Mat a his3-200, leu2-1, trp1-63, ura3::MLS1prom-mCherry-px, pex11::kanMX4		This study
CB371	Mat a his3-200, leu2-1, trp1-63, ura3-52, pex25::kanMX4	CB80 x BY4741*pex25*Δ	This study
CB372	Mat a his3-200, leu2-1, trp1-63, ura3-52, pex27::kanMX4	CB80 x BY4741*pex27*Δ	This study
CB374	Mat a his3-200, leu2-1, lys2Δ0, trp1-63, ura3-52, pex11::kanMX4, pex25::kanMX4	CB369 x BY4742*pex25*Δ	This study
CB375	Mat α his3-200, leu2-1, trp1-63, ura3-52, pex11::kanMX4, pex25::kanMX4	CB370 x BY4741*pex25*Δ	This study
CB376	Mat a his3-200, leu2-1, lys2Δ0, trp1-63, ura3-52, pex11::kanMX4, pex27::kanMX4	CB369 x BY4742*pex27*Δ	This study
CB417	Mat a his3-200, leu2-1, trp1-63, ura3-52, pex25::kanMX4, pex27::kanMX4	CB371 x BY4742*pex27*Δ	This study
CB419	Mat a his3-200, leu2-1, trp1-63, ura3-52, pex11::kanMX4, pex25::kanMX4, pex27::kanMX4	CB375 x BY4741*pex27*Δ	This study
CB545	Mat a his3-200, leu2-1, trp1-63, ura3::MLS1prom-mCherry-px, pex11::kanMX4, pex25::kanMX4, pex27::kanMX4		This study
CB547	Mat a his3-200, leu2-1, trp1-63, ura3-52, pex11::kanMX4, pex25::kanMX4, pex27::kanMX4, GalSprom-Pex3::natNT2		This study
CB537	Mat a his3-200, leu2-1, trp1-63, ura3-52 leu2Δ0::hphNT1-GAL-Sprom-yeGFP-px		This study
CB541	Mat a his3-200, leu2-1, trp1-63, ura3-52 inp2::hphNT1-GAL-Sprom-yeGFP-px		This study
CB542	Mat a his3-200, leu2-1, trp1-63, ura3-52, pex11::kanMX4 inp2::hphNT1-GAL-Sprom-yeGFP-px		This study
CB543	Mat a his3-200, leu2-1, trp1-63, ura3-52, pex25::kanMX4 inp2::hphNT1-GAL-Sprom-yeGFP-px		This study
CB544	Mat a his3-200, leu2-1, trp1-63, ura3-52, pex27::kanMX4 inp2::hphNT1-GAL-Sprom-yeGFP-px		This study
CB532	Mat a his3-200, leu2-1, lys2Δ0, trp1-63, ura3-52, pex11::kanMX4, pex25::kanMX4 inp2::hphNT1-GAL-Sprom-yeGFP-px		This study
CB533	Mat a his3-200, leu2-1, lys2Δ0, trp1-63, ura3-52, pex11::kanMX4, pex27::kanMX4 inp2::hphNT1-GAL-Sprom-yeGFP-px		This study
CB535	Mat a his3-200, leu2-1, trp1-63, ura3-52, pex25::kanMX4, pex27::kanMX4 inp2::hphNT1-GAL-Sprom-yeGFP-px		This study
CB536	Mat a his3-200, leu2-1, trp1-63, ura3-52, pex11::kanMX4, pex25::kanMX4, pex27::kanMX4 inp2::hphNT1-GAL-Sprom-yeGFP-px		This study
BY4741*pex25*Δ	Mat a, his3Δ1, leu2Δ0, lys2Δ0, ura3Δ0, pex25::kanMX4		Euroscarf
BY4741*pex27*Δ	Mat a, his3Δ1, leu2Δ0, lys2Δ0, ura3Δ0, pex27::kanMX4		Euroscarf
BY4742*pex25*Δ	Mat α, his3Δ1, leu2Δ0, lys2Δ0, ura3Δ0, pex25::kanMX4		Euroscarf
BY4742*pex27*Δ	Mat α, his3Δ1, leu2Δ0, lys2Δ0, ura3Δ0, pex27::kanMX4		Euroscarf
